# H_2_FPEF Score and Charlson Comorbidity Index as Independent Predictors of Cardiovascular Outcomes in Elderly Patients Undergoing Geriatric Rehabilitation

**DOI:** 10.1002/clc.70496

**Published:** 2026-10-16

**Authors:** Nicolas Dayer, Denise Auberson, Henri Lu, Elsa Costa, Hélène Krief, Sylvain Nguyen, Kristof Major, Wanda Bosshard, Roger Hullin, Pierre Monney

**Affiliations:** ^1^ Service of Cardiology University Hospital of Lausanne (CHUV) and University of Lausanne (Unil) Lausanne Switzerland; ^2^ Service of Cardiology Centre Hospitalier du Valais Romand (CHVR) Sion Switzerland; ^3^ Service of Geriatrics University Hospital of Lausanne (CHUV) and University of Lausanne (Unil) Lausanne Switzerland; ^4^ Service of Geriatrics Riviera‐Chablais Hospital (HRC) Rennaz Switzerland

**Keywords:** Charlson comorbidity index, functional dependency, H_2_FPEF score, heart failure with preserved ejection fraction, HFpEF, prognostic score

## Abstract

**Background:**

Heart failure with preserved ejection fraction (HFpEF) is difficult to diagnose in older adults. The H_2_FPEF score estimates the likelihood of HFpEF and has been associated with adverse prognosis, but its performance has not been evaluated in geriatric inpatients with a high comorbidity burden.

**Hypothesis:**

We hypothesized that the H_2_FPEF score predicts adverse outcomes in geriatric inpatients independently of comorbidity burden.

**Methods:**

This prospective cohort included consecutive patients admitted to a geriatric rehabilitation unit. All patients underwent functional and echocardiographic assessment at admission. The ability of the H_2_FPEF score, the Charlson comorbidity index (CCI), and their combination to predict 1‐year all‐cause mortality (ACM) or cardiovascular rehospitalization (CR) was evaluated.

**Results:**

Among 302 patients (median age 83 years, 40% men), 24% had a H_2_FPEF score ≥ 6 indicating high HFpEF probability and 42% had a CCI > 3 indicating high comorbidity burden. The H_2_FPEF score correlated with CCI (*β* = 0.27, *p* < 0.01) and functional disability (lower Instrumental Activities of Daily Living score; *β* = −0.17, *p* = 0.003), but not with cognitive impairment. Both a high H_2_FPEF score and high CCI were associated with a higher risk of the combined outcome of 1‐year ACM or CR (adjusted HR 2.89 [95% CI 1.63–5.10], *p* < 0.001 and adjusted HR 2.66 [95% CI 1.67–4.21], *p* < 0.001, respectively), with no interaction between them (*p* = 0.63). Patients with both high‐risk scores had the highest risk (adjusted HR 4.66 [95% CI 2.54–8.57], *p* < 0.001).

**Conclusions:**

In geriatric inpatients, a high H_2_FPEF score is common and independently identifies patients at increased risk of adverse cardiovascular outcomes at 1 year.

## Clinical Perspectives

- In older inpatients with preserved LVEF admitted to geriatric rehabilitation, one in four had a high H₂FPEF score, underscoring a substantial burden of likely HFpEF in this setting.
- The combination of a high H₂FPEF score and a high Charlson Comorbidity Index independently identified patients at higher 1‐year risk of death or cardiovascular rehospitalization, this highlights the complementary prognostic value of both scoring systems.
- The coexistence of high H₂FPEF score and high comorbidity burden was associated with a 4‐5‐fold higher hazard of adverse outcome, suggesting that combining these two simple scores may help clinicians detect a very high‐risk subgroup requiring intensified and tailored management.

## Graphical abstract

In older patients undergoing geriatric rehabilitation with preserved LVEF, a high H_2_FPEF score (≥6) independently predicted 1‐year death or cardiovascular rehospitalization. Combining H_2_FPEF with the Charlson comorbidity index identified a very‐high‐risk subgroup, with a 4.66‐fold higher risk of adverse outcomes.

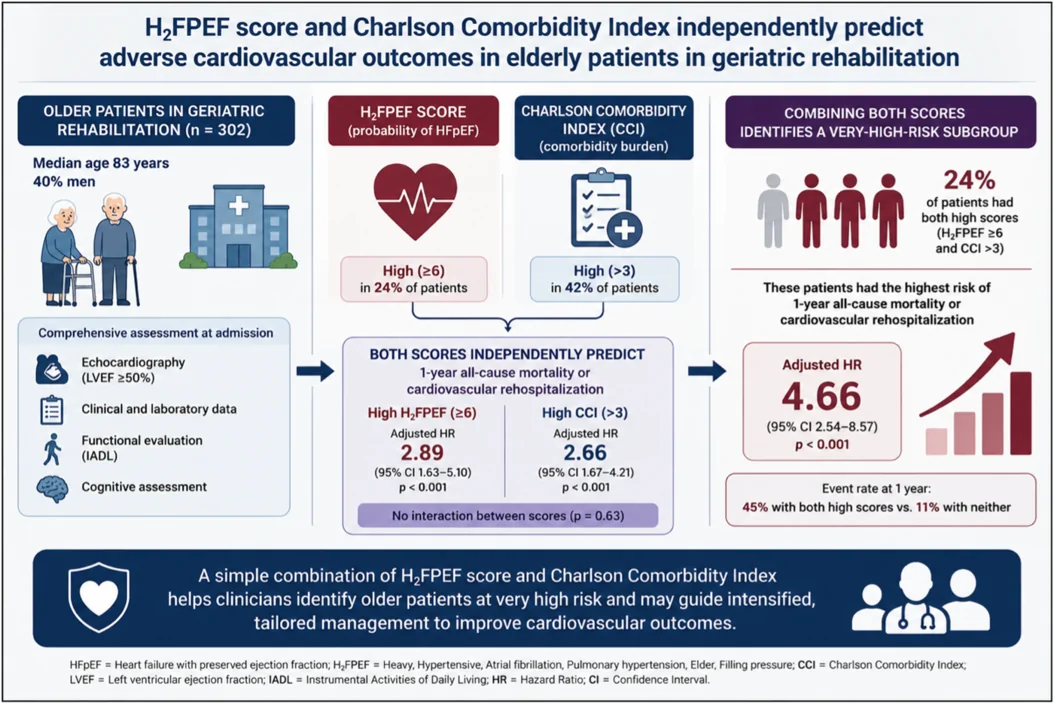

**Abbreviations**
AF: atrial fibrillation
BADL: basic activities of daily living
CCI: Charlson comorbidity index
EACVI: European association of cardiovascular imaging
FIM: functional independence measure
GLS: global longitudinal strain
HFpEF: heart failure with preserved ejection fraction
IADV: instrumental activities of daily living
IQR: interquartile range
LA: left atrium
LARS: left atrial reservoir strain
LVEF: left ventricular ejection fraction
MMSE: mini mental status exam
TAPSE: tricuspid annular plan systolic excursion
TTE: transthoracic echocardiography

## 1 Introduction

The prevalence of heart failure with preserved ejection fraction (HFpEF) is high among older adults, accounting for at least two thirds of heart failure (HF) cases in the community [1, 2, 3, 4]. HFpEF diagnosis is based on the association of signs and symptoms of HF, a left ventricular ejection fraction (LVEF) ≥ 50%, and on the objective evidence of cardiac structural or functional abnormality consistent with the presence of LV diastolic dysfunction and raised filling pressures [5]. To guide the clinician, relatively complex stepwise algorithms have been proposed, combining clinical and echocardiographic data, natriuretic peptide dosages and echocardiographic diastolic stress test results [6]. Simpler approaches include the HFpEF‐ABA score based on age, body mass index and the presence of atrial fibrillation (AF) [7], and the H_2_FPEF score, the score we chose to use for the present study as it also incorporates important echocardiographic data. It is a simple 9‐points score specifically developed to estimate the likelihood of HFpEF in patients with unexplained exertional dyspnea, a score ≥ 6 being associated with *a* > 90% probability (Supporting Information 1) [8]. In patients over 65 years, the score has demonstrated a sensitivity of 77% and a specificity of 97% [9]. Subsequent studies established H_2_FPEF score not only as a diagnostic tool, but also as a valuable metric to identify patients with more severe forms of HFpEF, to screen at‐risk patients for preclinical HFpEF, or to estimate the prognosis in terms of all‐cause mortality (ACM) or rehospitalization [10, 11].

Older adults admitted for a geriatric in‐patient rehabilitation program after surgery or acute medical hospitalization are at substantial risk of HFpEF due to older age and a high prevalence of comorbidities. Moreover, the symptom of dyspnea may not be specific for HF in such a population [12]. We therefore hypothesized that the systematic use of the H_2_FPEF score in patients with LVEF ≥ 50% attending an inpatient geriatric rehabilitation program should identify patients with high likelihood of HFpEF diagnosis and higher risk of cardiovascular endpoints, independently of the comorbidity load and the presence of a self‐reported symptom of dyspnea.

The first aim of this study was to assess the 1‐year prognosis in terms of a composite end‐point of ACM and rehospitalization for cardiovascular causes based on the H_2_FPEF score in a population of older (> 65 years) patients with LVEF ≥ 50% undergoing an inpatient rehabilitation program, as well as its relation with the comorbidity burden assessed with the Charlson comorbidity index (CCI). The second aim was to describe the prevalence of patients with an H_2_FPEF score ≥ 6 and to assess the relation of the H_2_FPEF score with indirect markers of HF (including dyspnea, echocardiographic evidence of elevated LV filling pressure and previous hospitalizations for HF) and with markers of comorbidity and functional impairment (including CCI, Basic Activities of Daily Living [BADL], Instrumental Activities of Daily Living [IADL] score and Functional Independence Measure [FIM] score).

## 2 Methods

### 2.1 Study Design and Setting

This observational, prospective, single‐center study was conducted at Lausanne University Hospital (Switzerland) from November 2021 to November 2022, approved by the Ethics Committee (CER‐VD 2021‐01693). All included patients provided written informed consent.

### 2.2 Participants and Data Collection

Consecutive patients aged > 65 years admitted for in‐patient geriatric rehabilitation after acute hospitalization, either for a medical condition (e.g., infection, HF, acute illness) or after a surgical intervention (most commonly non‐elective orthopedic surgery), were invited to participate and to undergo a research transthoracic echocardiography (TTE). Exclusion criteria were the absence of consent, LVEF < 50%, patients lost at 1‐year follow‐up, and missing echocardiographic parameters for H_2_FPEF score assessment. Baseline data were collected at admission by the main investigators (N.D., K.M., and D.A.) either by direct interview or by review of the charts, including medical history, actual symptoms, vital signs, ECG, and selected laboratory values. Functional geriatric evaluation scores were collected in all patients at admission by geriatricians, including BADL, IADL, FIM, and Mini‐Mental State Examination (MMSE). However, MMSE was not repeated if it was already performed in the past 3 months, during the acute admission (Supporting Information 2). Comorbidity burden was assessed in all patients using the CCI, and a CCI > 3 was considered high comorbidity load [13].

### 2.3 Echocardiographic Assessment and H_2_FPEF Score Calculation

TTE was performed in all patients during the first week of admission by a certified technician on an EPIQ CVx machine (Philips, the Netherlands) and analyzed offline by a board‐certified cardiologist. A comprehensive examination including global longitudinal strain (GLS) and left atrial reservoir strain (LARS) was performed according to the current guidelines [14, 15, 16, 17]. Strain analysis was only performed on images of sufficient technical quality. Left ventricular filling pressures and diastolic function were assessed according to current guidelines [18, 19, 20]. Combining the clinical and echocardiographic data, the H_2_FPEF score was calculated for all patients, and the cohort was stratified into three classes of HFpEF probability: H_2_FPEF score ≤ 2 (low risk), H_2_FPEF 3‐5 (intermediate risk), and H_2_FPEF ≥ 6 (high risk) [8].

### 2.4 Follow‐up and End‐Points

Follow‐up at 1 year was complete for all patients. Vital status and hospitalization data were collected by telephone based on patient or proxy report and review of the available discharge documents. The primary endpoint was a composite of ACM or cardiovascular rehospitalization (CR), with censoring after the occurrence of the first event. Secondary endpoints were ACM, rehospitalization for cardiovascular causes, and rehospitalization for non‐cardiovascular causes, respectively.

### 2.5 Statistical Analyses

Continuous variables were expressed as median with interquartile range (IQR) and compared across the H_2_FPEF probability classes using the Kruskal–Wallis test. Categorical variables were expressed as counts and percentages and were compared across the 3 H_2_FPEF classes using a Chi_2_ test. A *p* for trend was calculated for all variables according to Cuzick [21]. The correlations between the H_2_FPEF score and either dyspnea, previous HF hospitalization, or evidence of elevated filling pressures were first assessed by bi‐variate logistic regression and then by logistic regression with adjustment for age and sex. Similarly, the correlations between the H_2_FPEF score and either IADL score, FIM score, or CCI were first assessed by bi‐variate linear regression and then by linear regression with adjustment for age and sex. The combined outcome of ACM and rehospitalization for cardiovascular cause according to H_2_FPEF score groups were displayed using Kaplan−Meier curves and compared using the log‐rank test. The association of increasing H_2_FPEF probability classes, respectively high CCI, with the combined outcome were assessed using bivariable and multivariable Cox proportional hazards models (results reported as hazard ratios with 95% confidence intervals). Multivariable analyses were adjusted for age and sex, and interaction was assessed between H_2_FPEF high probability class and the presence of dyspnea, previous HF hospitalization and/or high CCI. A two‐sided *p* value < 0.05 was considered statistically significant. Analyses were performed in STATA 19 (StataCorp LLC, College Station, TX, USA).

## 3 Results

### 3.1 Study Population

A total of 1022 patients were screened for the study, and 302 patients were finally included for analysis after applying the exclusion criteria (Figure 1). Baseline characteristics are summarized in the Table 1. Median age was 83 [77–87] years and 40% were men; dyspnea was reported in 78 (26%), 48 (16%) had AF at the time of TTE and 33 (11%) had previous myocardial infarction. 84 (28%) had previous admission for HF, 110 (37%) moderate‐to‐severe renal failure and 74 (25%) chronic pulmonary disease. The median number of different medications was 7 [5, 6, 7, 8, 9] and a CCI > 3 was present in 128 (42%). Functional assessment revealed reduced independence (BADL score = 5.5 [4–6], IADL score = 4 [2–6]) and 83 patients (27%) had cognitive impairment at admission (MMSE ≤ 24). By TTE, median LVEF was 65 [61–68]%; 37 (12%) had segmental wall motion abnormalities and 48 (16%) left ventricular hypertrophy. Median GLS was 20.6 [18.1–23.0]% and median LARS was 25.2 [14.8–33.3]%. LV filling pressures were elevated by TTE in 112 (37%), normal in 156 (52%) and indeterminate in 34 (11%). Moderate or severe aortic stenosis was present in 31 (10%), ≥ 2+ mitral regurgitation in 14 (5%), and ≥ 2+ tricuspid regurgitation in 36 (12%).

**Figure 1:**
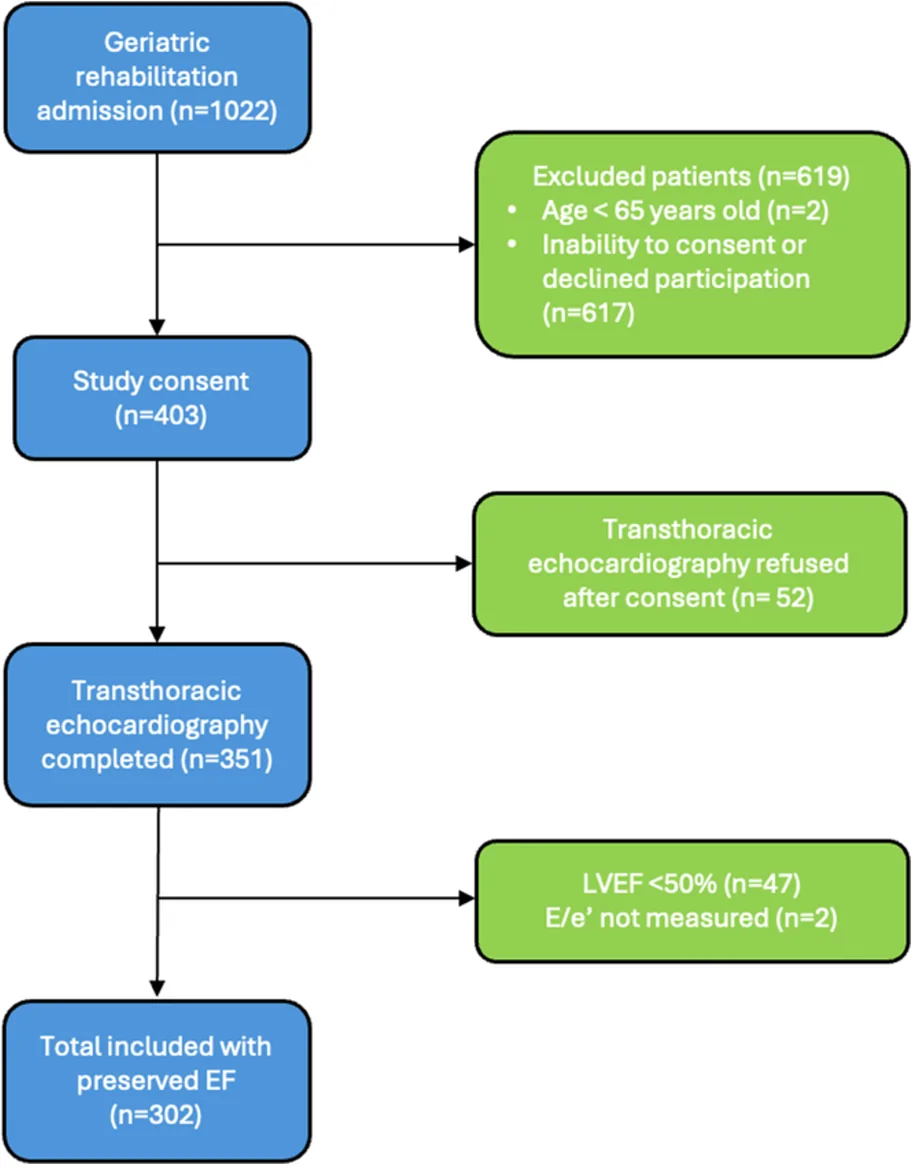
Flowchart. LVEF, left ventricular ejection fraction.

**Table 1:** Baseline characteristics.

Variable	Unit	*N*	Total population (*n* = 302)
Age	Years	302	83 [77–87]
Male sex	*n* (%)	302	121 (40%)
Body mass index	kg/m2	302	24.6 [21.5–28.6]
Systolic blood pressure	mmHg	302	134 [118–145]
Diastolic blood pressure	mmHg	302	69 [60–78]
Heart rate	bpm	302	72 [66–82]
Atrial fibrillation	*n* (%)	302	48 (16%)
Dyspnea	*n* (%)	302	78 (26%)
Previous heart failure episode	*n* (%)	302	115 (38%)
Previous heart failure hospitalization	*n* (%)	298	84 (28%)
Previous myocardial infarct	*n* (%)	302	33 (11%)
Diabetes	*n* (%)	302	80 (26%)
Cognitive impairment (MMSE < 24)	*n* (%)	302	83 (27%)
Kidney failure (eGFR < 60 mL/min)	*n* (%)	301	110 (37%)
Chronic pulmonary disease	*n* (%)	302	74 (25%)
Number of medications	*n*	296	7 [5–9]
Total Charlson comorbidity index	0–37	302	3 [2–5]
High comorbidity (CCI > 3)	*n* (%)	302	128 (42%)
BADL score	0–6	302	5.5 [4–6]
IADL score	0–8	302	4 [2–6]
MMSE score	0–30	260	27 [24–28]
FIM score	18–126	300	101 [88–109]
Tinetti score	0–28	300	19 [15–23]
Body mass index > 30 kg/m^2^	*n* (%)	302	50 (17%)
≥ 2 anti‐hypertensive medications	*n* (%)	302	94 (31%)
Atrial fibrillation (history of)	*n* (%)	302	112 (37%)
High pulmonary pressure (echo)	*n* (%)	290	69 (24%)
Age > 60 years	*n* (%)	302	302 (100%)
E/e' ratio > 9	*n* (%)	302	213 (71%)
Total H_2_FPEF score	0–9	302	3 [2–5]
Left ventricular ejection fraction (LVEF)	%	287	65 [61–68]
Left ventricular hypertrophy (LVH)	*n* (%)	291	48 (16%)
Regional wall motion abnormalities	*n* (%)	299	37 (22%)
Global longitudinal strain (GLS)	%	188	20.6 [18.1–23.0]
Left atrial volume index	mL/m^2^	219	34.1 [26.1–42.6]
Left atrial reservoir strain	%	252	25 [15–33]
E/e' ratio (average)	—	302	10.7 [8.5–13.6]
Elevated filling pressures	*n* (%)	302	112 (37%)
Tricuspid annular plane systolic excursion (TAPSE)	mm	293	21 [19–24]
Moderate or severe aortic stenosis (AS)	*n* (%)	299	31 (10%)
≥ 2+ aortic regurgitation (AR)	*n* (%)	301	8 (3%)
≥ 2+ mitral regurgitation (MR)	*n* (%)	300	14 (5%)
≥ 2+ tricuspid regurgitation (TR)	*n* (%)	300	36 (12%)

*Note:* Data are presented as mean ± standard deviation, (%) or median and interquartile range.

Abbreviations: BADL, basic activities of daily living; CCI, Charlson comorbidity index; eGFR, estimated glomerular filtration rate; FIM, functional independence measurement; IADL, instrumental activities of daily living; MMSE, mini mental state exam.

### 3.2 Patient Stratification According to H_2_FPEF Risk Class

The comparison of baseline characteristics according to different likelihood to HFpEF diagnosis is summarized in the Table 2. Among the 302 patients, 122 (40%) were low risk, 109 (36%) were intermediate risk, and 71 (24%) were high risk class. Patients with high‐risk H_2_FPEF were older and had more frequently AF and/or a history of HF hospitalization. A higher comorbidity burden was reflected by a higher CCI and a higher median number of medications. Echocardiographic assessment indicated that patients with high‐risk H_2_FPEF had a slightly lower LVEF and lower GLS. They also had larger LA with markedly lower LARS. Right ventricular function was also lower as reflected by lower tricuspid annular plane systolic excursion (TAPSE).

**Table 2:** Baseline characteristics according to H_2_FPEF risk class.

Variable	Unit	*N*	H_2_FPEF < 3*n* = 122	H_2_FPEF 3−5 *n* = 109	H_2_FPEF ≥ 6 *n* = 71	*p* for trend
Age	Years	302	80 [75–86]	83 [76–87]	86 [80–90]	0.0001
Male sex	*n* (%)	302	42 (34%)	52 (48%)	27 (38%)	0.41
Body mass index	kg/m2	302	23.9 [21.3–26.3]	26.1 [22.1–30.8]	24.6 [21.5–30.5]	0.01
Systolic blood pressure	mmHg	302	130 [115–143]	135 [123–146]	136 [123–148]	0.018
Diastolic blood pressure	mmHg	302	69 [60–78]	68 [61–77]	70 [58–79]	0.92
Heart rate	bpm	302	72 [66–80]	72 [67–82]	75 [65–85]	0.12
Atrial fibrillation	*n* (%)	302	0 (0%)	15 (14%)	33 (46%)	< 0.0001
Dyspnea	*n* (%)	302	24 (20%)	30 (28%)	24 (34%)	0.027
Previous heart failure episode	*n* (%)	302	24 (20%)	40 (37%)	51 (72%)	< 0.0001
Previous HF hospitalization	*n* (%)	298	13 (11%)	29 (27%)	42 (61%)	< 0.0001
Previous myocardial infarct	*n* (%)	302	8 (l7%)	14 (13%)	11 (15%)	0.04
Diabetes	*n* (%)	302	22 (18%)	39 (36%)	19 (27%)	0.08
Cognitive impairment (MMSE < 24)	*n* (%)	302	30 (25%)	32 (29%)	21 (30%)	0.41
Kidney failure (eGFR < 60 mL/min)	*n* (%)	301	29 (24%)	35 (32%)	46 (66%)	< 0.0001
Chronic pulmonary disease	*n* (%)	302	23 (19%)	32 (29%)	19 (27%)	0.15
Number of medications	*n*	296	6 [4–8]	8 [6–10]	8 [6–10]	< 0.0001
Total Charlson comorbidity index	0–37	302	2 [1–4]	3 [2–6]	4 [3–6]	< 0.0001
High comorbidity (CCI > 3)	*n* (%)	302	39 (32%)	47 (43%)	42 (59%)	0.0002
BADL score	0–6	302	6 [5–6]	5 [4–6]	5 [4–6]	0.034
IADL score	0–8	302	5 [3–7]	4 [2–6]	4 [2–5]	0.002
MMSE score	0–30	260	27 [24–28]	27 [24–28]	26 [24–28]	0.30
FIM score	18–126	300	104 [92–111]	100 [87–106]	97 [86–106]	0.005
Body mass index > 30 kg/m^2^	*n* (%)	302	0 (0%)	33 (30%)	17 (24%)	< 0.0001
≥ 2 anti‐hypertensive medications	*n* (%)	302	10 (8%)	45 (41%)	39 (54%)	< 0.0001
Atrial fibrillation (history of)	*n* (%)	302	0 (0%)	41 (38%)	71 (100%)	< 0.0001
High pulmonary pressure (echo)	*n* (%)	290	3 (3%)	20 (19%)	46 (69%)	< 0.0001
Age > 60 years	*n* (%)	302	122 (100%)	109 (100%)	71 (100%)	—
E/e' ratio > 9	*n* (%)	302	68 (56%)	77 (70%)	68 (96%)	< 0.0001
Total H_2_FPEF score	0–9	302	2 [1–2]	4 [3–5]	6 [6–7]	—
Left ventricular ejection fraction	%	287	65 [63–69]	64 [60–68]	62 [57–67]	0.002
Left ventricular hypertrophy	*n* (%)	291	12 (10%)	17 (17%)	19 (28%)	0.0016
Regional wall motion abnormalities	*n* (%)	299	11 (9%)	11 (10%)	15 (21%)	0.02
Global longitudinal strain	%	188	21.3 [19.6–23.7]	20.6 [18.1–22.7]	18.1 [15.7–20.5]	< 0.0001
Left atrial volume index	mL/m2	219	29.3 [24.1–35.7]	34.7 [24.2–42.9]	46.0 [37.3–54.2]	< 0.0001
Left atrial reservoir strain	%	252	28 [24–37]	27 [17–34]	13 [9–17]	< 0.0001
E/e' ratio	—	302	9.3 [7.6–11.6]	10.6 [8.7–13.0]	13.8 [11.1–17.]	< 0.0001
Elevated filling pressures	*n* (%)	302	32 (26%)	40 (37%)	40 (56%)	< 0.0001
TAPSE	mm	293	22 [20–25]	22 [19–24]	18 [15–21]	< 0.0001
Moderate or severe aortic stenosis	*n* (%)	299	8 (7%)	12 (11%)	11 (16%)	0.036
≥2+ aortic regurgitation	*n* (%)	301	5 (4%)	1 (1%)	2 (3%)	0.46
≥2+ mitral regurgitation	*n* (%)	300	5 (4%)	2 (2%)	7 (10%)	0.12
≥2+ tricuspid regurgitation	*n* (%)	300	2 (2%)	9 (8%)	25 (36%)	< 0.0001

*Note:* Data are presented as mean ± standard deviation, (%) or median and interquartile range.

Abbreviations: BADL, basic activities of daily living; CCI, Charlson comorbidity index; eGFR, estimated glomerular filtration rate; FIM, functional independence measurement; IADL, instrumental activities of daily living; MMSE, mini mental state exam.

### 3.3 One‐Year Prognostic Value of High H_2_FPEF Risk Class and Relation With Comorbidities

The median follow‐up duration was 387 [372−410] days. The outcome results according to H_2_FPEF risk classes are summarized in the Table 3 and in the Figure 2. The primary endpoint of ACM or rehospitalization significantly increased across the 3 H_2_FPEF groups from 17% in low‐risk to 44% in the high‐risk H_2_FPEF class (*p* = 0.0001). Compared with patients in low‐risk H_2_FPEF class, those in high‐risk class had higher risk of the primary endpoint after adjustment for age and sex (adjusted HR 2.89 [95%‐CI 1.63–5.10], *p* < 0.001). No evidence of effect modification was found for dyspnea or history of HF hospitalization (p for interaction 0.74 and 0.78, respectively). Likewise, compared to low CCI, a high CCI (>3 points) was associated with the primary endpoint with an adjusted HR of 2.66 [95%‐CI 1.67−4.21], *p* < 0.001. No interaction was found between a CCI > 3 and an H_2_FPEF score ≥ 6 for the association with the primary endpoint in multivariate analysis (*p* for interaction 0.63).

**Table 3:** Outcomes according to the H_2_FPEF risk class.

	Low risk H2FPEF < 3	Intermediate risk H2FPEF 3‐5	High‐risk H2FPEF ≥ 6
	*n* = 122	*n* = 109	*n* = 71
CV outcomes			
*All‐cause mortality or rehospitalization for cardiovascular cause*			
*n*	21	29	31
Unadjusted (95% CI)	1 (reference)	1.62 (0.93–2.85)	3.20 (1.84–5.58)
Adjusted* (95% CI)	1 (reference)	1.51 (0.85–2.66)	2.89 (1.63–5.10)
*All‐cause mortality*			
*n*	13	19	20
Unadjusted (95% CI)	1 (reference)	1.66 (0.82–3.37)	3.00 (1.49–6.03)
Adjusted* (95% CI)	1 (reference)	1.53 (0.75–3.13)	2.63 (1.28–5.39)
*Rehospitalization for cardiovascular cause*			
*n*	9	13	18
Unadjusted (95% CI)	1 (reference)	1.66 (0.71–3.89)	4.15 (1.86–9.24)
Adjusted* (95% CI)	1 (reference)	1.56 (0.66–3.67)	3.86 (1.70–8.80)
*Rehospitalization for non‐cardiovascular cause*			
*n*	53	55	34
Unadjusted (95% CI)	1 (reference)	1.15 (0.79–1.68)	1.21 (0.80–1.87)
Adjusted* (95% CI)	1 (reference)	1.08 (0.73–1.58)	1.24 (0.78–1.94)

*Note:* * Adjusted for age and sex. Values are hazard ratios (95% CI), reference = low H_2_FPEF group.

**Figure 2:**
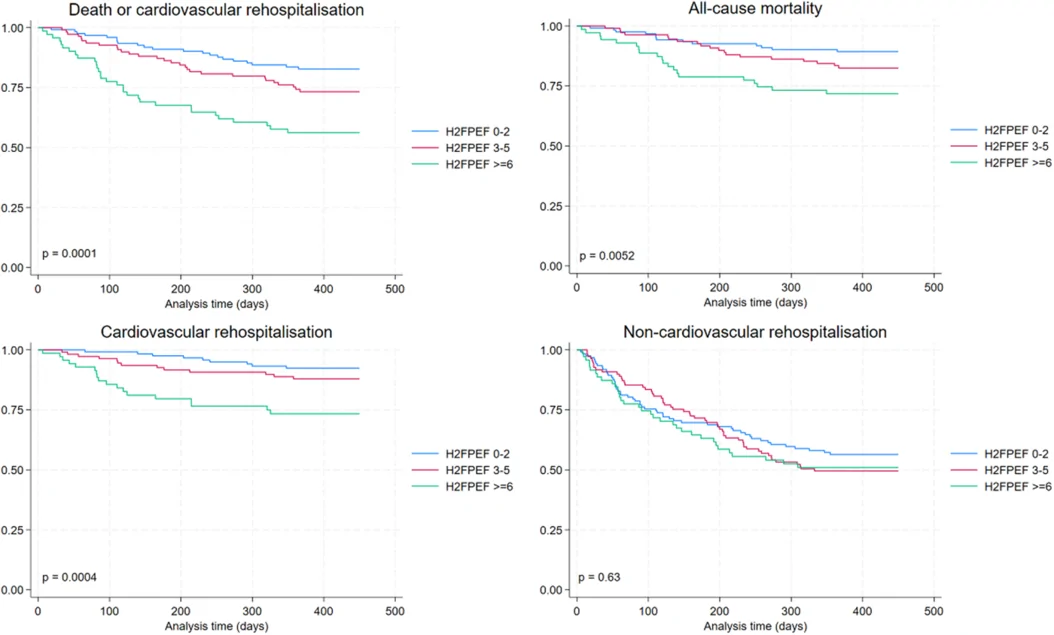
Clinical outcomes according to H_2_FPEF score. Kaplan−Meier curves for death or cardiovascular rehospitalization, all‐cause mortality, cardiovascular rehospitalization and non‐cardiovascular rehospitalization over follow‐up, stratified by H_2_FPEF score (0–2, 3−5, ≥ 6) with log‐rank *p* values shown in each panel.

Figure 3 illustrates the independent association of high CCI and high H_2_FPEF score with the primary endpoint. Compared to patients with a CCI ≤ 3 and a H_2_FPEF score < 6, patients with only H_2_FPEF score ≥ 6 had an adjusted HR of 2.28 (95%‐CI 1.02−5.07, *p* = 0.043), those with only CCI > 3 an adjusted HR of 2.58 (95%‐CI 1.46–4.56, *p* = 0.001), and those with both H_2_FPEF score ≥ 6 and CCI > 3 an adjusted HR of 4.66 (95%‐CI 2.54–8.57, *p* < 0.001).

**Figure 3:**
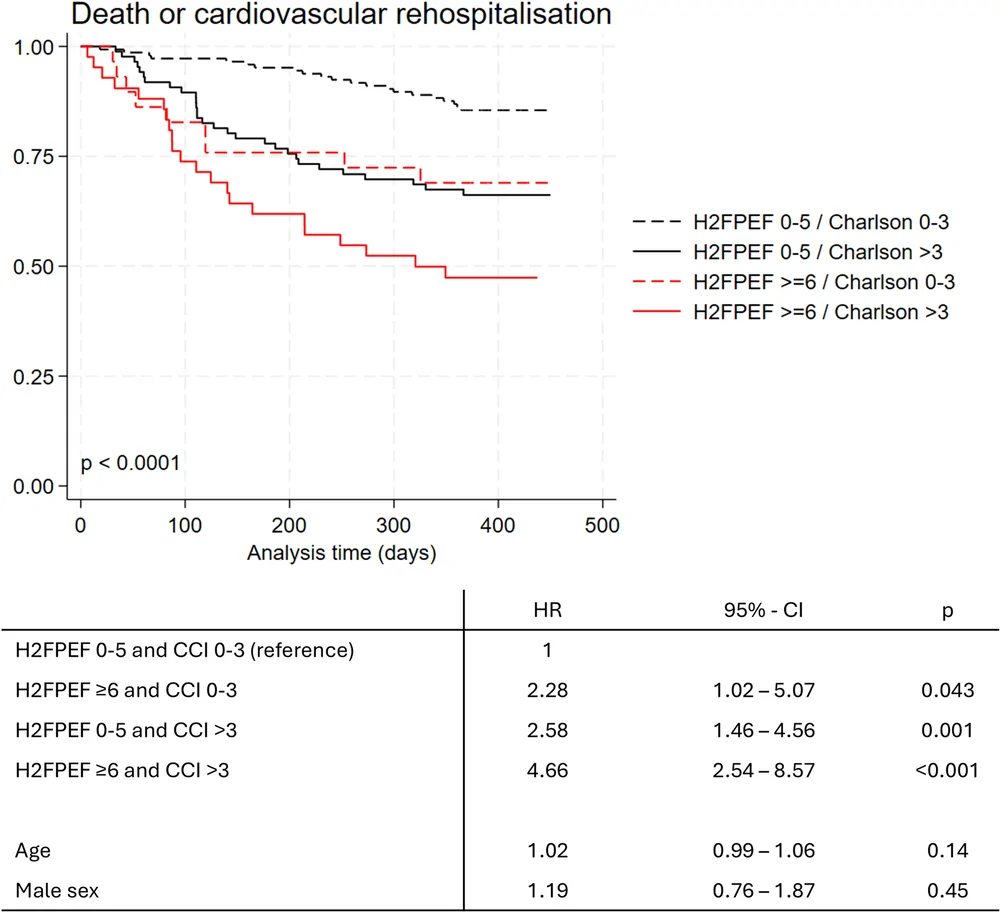
Outcomes according to Charlson comorbidity index and H_2_FPEF score. Kaplan−Meier curves for the composite endpoint of death or cardiovascular rehospitalization over follow‐up, stratified by H_2_FPEF score (0–5 vs. ≥ 6) and Charlson comorbidity index (0‐3 vs. ≥ 3), global log‐rank *p* < 0.0001.

Compared to low‐risk H_2_FPEF class, the high‐risk H_2_FPEF class had a higher risk of the secondary endpoints of ACM (adjusted HR 2.63 [95%‐CI 1.28–5.39], *p* = 0.008) and rehospitalization for cardiovascular cause (adjusted HR 3.86 [95%‐CI 1.70–8.80], *p* = 0.001), but not of rehospitalizations for non‐cardiovascular causes (adjusted HR 1.24 [95%‐CI 0.78–1.94], *p* = 0.36). Conversely, a high CCI was also significantly associated with ACM (adjusted HR 4.30 [95%‐CI 2.99–8.04], *p* < 0.001) and non‐CR (adjusted HR 2.14 [95%‐CI 1.53–2.99], *p* < 0.001), but not with CR (adjusted HR 1.37 [95%‐CI 0.73–2.59], *p* = 0.33).

### 3.4 Association Between H_2_FPEF Risk Class and Surrogate Markers of HF and Functional Disability

The prevalence of dyspnea significantly increased from 20% in the low risk to 34% in the high‐risk class (*p* for trend 0.03). By logistic regression adjusted for age and sex, the H_2_FPEF score had a significant correlation with dyspnea (OR 1.23, 95%‐CI 1.08–1.40, *p* = 0.002). A similar stepwise increase in history of previous HF hospitalization was observed between low and high‐risk H_2_FPEF classes (from 11% to 61%, *p* for trend < 0.0001), and there was a significant adjusted correlation between H_2_FPEF score and history of HF hospitalization (OR 1.71, 95%‐CI 1.47–1.99, *p* < 0.001). Expectedly, elevated filling pressures estimated by TTE were present in 26% of low‐risk and 56% of high‐risk H_2_FPEF class patients, and the H_2_FPEF score had a significant adjusted correlation with elevated filling pressures (OR 1.27, 95%‐CI 1.21–1.43, *p* < 0.001). A significant linear correlation was observed between H_2_FPEF score and the CCI after adjustment for age and sex (*β* = 0.27, *p* < 0.01), as well as an inverse adjusted linear correlation of H_2_FPEF score with the IADL score (*β* = −0.17, *p* = 0.003). An inverse linear correlation was also found between H_2_FPEF score and FIM in bivariate analysis, which was no more significant after adjustment for age and sex (*β* = −0.08, *p* = 0.14).

## 4 Discussion

In a population with a median age of 83 years undergoing inpatient rehabilitation after acute hospitalization, our study provided several important clinical findings. First, one in four patients had a H_2_FPEF score ≥ 6, which had been associated with *a* > 90% probability of HFpEF diagnosis [8]. Second, the presence of a high‐risk H_2_FPEF score in older patients had a strong prognostic value as it was associated with a higher risk of ACM or CR after 1 year, even after adjustment for age and sex. Third, despite a significant correlation between the H_2_FPEF score and the CCI, both scores were independently (p for interaction 0.63) associated with the 1‐year primary endpoint. Fourth, although higher H_2_FPEF score was associated with a lower grade of independence in the activities of daily living, the cognitive performance remained equally preserved in patients with high and low risk H_2_FPEF scores.

HFpEF is a particularly challenging diagnosis due to the low specificity of dyspnea and/or peripheral oedema, and the multiplicity of coexisting comorbidities, such as AF, obesity, metabolic syndrome, sarcopenia, diabetes mellitus, hypertension and chronic kidney disease [1, 12]. Moreover, it is recognized that typical HF signs may not be present on physical examination in the early stages of HFpEF [5], and the use of scores and biomarkers is recommended to improve detection. The H_2_FPEF score was derived and validated against invasive hemodynamic measurements in a relatively younger cohort of dyspneic patients with a mean age of 64 years, including two‐thirds with HFpEF diagnosis and one‐third with non‐cardiac dyspnea [8]. Therefore, its external validity in our cohort of older inpatients (median age 83 years) may be questioned. Yet, in the ARIC study, (mean age 75 years), among the 13% of the cohort complaining of unexplained dyspnea, a high‐risk H_2_FPEF score was present in 35% and their prognosis was comparable to the 10% of the cohort with a confirmed HFpEF diagnosis [22], supporting the validity of the score in older patients. Applied to a subset of the TOPCAT HFpEF trial, the H_2_FPEF score was shown to correlate with the risk of cardiovascular adverse events. However, the score was only partially and non‐significantly associated with natriuretic peptides and a high proportion (25%–41%) of those patients with confirmed HFpEF had H_2_FPEF scores indicating a low (< 55%) HFpEF probability. These findings emphasized the need to better understand the validity of this score in larger heterogenous populations [23]. In our study, we applied the H_2_FPEF score to a specific but heterogenous population of very old patients undergoing inpatient rehabilitation program after acute hospitalization for various indications, including orthopedic surgery, infections, or decompensated HF. In that population, a high proportion of 24% of patients had a H_2_FPEF score ≥ 6 indicating a high risk of HFpEF. Although neither invasive hemodynamic measurements nor natriuretic peptides were systematically available, several findings supported the presence of a significant cardiac involvement in the high‐risk H_2_FPEF group, including a high prevalence of previous HF episodes (72%) or hospitalizations (61%), a high prevalence of current (46%) or history of (100%) AF, lower LARS and lower TAPSE, as well as a high proportion of patients with elevated LV filling pressures (56%). Higher H_2_FPEF score was also associated with slightly lower LVEF and lower LV GLS, suggesting that subtle systolic dysfunction may contribute to HFpEF pathophysiology in older inpatients, in line with Dunlay et al. [24].

As reported in several studies, the H_2_FPEF score has a significant prognostic value [10, 11, 23]. This was also the case in our cohort, where patients with H_2_FPEF ≥ 6 had a two to threefold higher risk of death or CR at 1 year compared to patients with H_2_FPEF < 3. Furthermore, our population was characterized by polypharmacy and a high burden of comorbidity, as often seen in geriatric post‐acute care rehabilitation, and a high CCI (>3 points) was present in 42% of the patients. Like the H_2_FPEF score, a high CCI was associated with a two‐ to threefold higher risk to reach the primary endpoint at 1 year. Although both scores showed significant correlation, there was no interaction between the two scores for the association with the primary endpoint and, in patients with both an H_2_FPEF score ≥ 6 and a CCI > 3, the risk of adverse outcome increased to 4−5‐fold. Moreover, while H_2_FPEF ≥ 6 was associated with CRs, a CCI > 3 was associated with non‐CRs, suggesting that H_2_FPEF score and CCI should relate to different aspects—cardiac versus non‐cardiac, of adverse prognosis in our population. These findings illustrate the added value for clinical usefulness of both scores in guiding tailored management: the H_2_FPEF score to identify patients who may benefit from targeted cardiac interventions, and the CCI those in whom broader geriatric strategies addressing polypharmacy, frailty and non‐cardiac comorbidities may be warranted.

AF appears to play a particularly important role for outcome prediction in this population. It was highly prevalent in patients with a high‐risk H2FPEF score and had been shown to be associated with adverse cardiovascular outcome in several studies. In a propensity score matched analysis of the TOPCAT Americas trial including patients with HFpEF, AF was consistently associated with a significantly higher risk of cardiovascular death, cardiovascular hospitalizations and HF hospitalizations over of median follow‐up of 2.9 years [25]. In a post‐hoc analysis of the AFFIRM trial, which randomized patients with recent AF to either rate or rhythm control strategy, HF occurrence was more frequent with a rate control strategy, and HF development was associated with a 3−4 times higher risk of cardiovascular mortality and cardiovascular hospitalizations at 5 years [26]. Interestingly, while it has a strong weight in the H2FPEF score, AF is not part of the CCI calculation; this could in part explain the specific association of H2FPEF score with cardiovascular adverse outcomes while CCI was merely associated with non‐cardiovascular adverse outcomes.

An overlap between HF and geriatric comorbidities is frequent among older patients, and the presence of physical, social, or cognitive limitations negatively impacts prognosis [27, 28, 29]. In our cohort, we only observed a significant correlation between the H_2_FPEF score and impaired autonomy (IADL score). However, although 27% of the cohort had cognitive impairment, no association was found between MMSE score and the H_2_FPEF score. In elderly patients with HF, a holistic approach is recommended, and admission to a rehabilitation unit offers an opportunity for interventions that may improve quality of life and outcomes. This may include exercise and physical rehabilitation, nutritional support, and cognitive support [27]. Whereas geriatricians are proficient to detect physical, social, or cognitive frailty in HF patients, HFpEF diagnosis remains a challenge in elderly patients. According to a recent survey, compared to HF specialists, both cardiologists and non‐cardiologists were significantly less likely to use specific HFpEF scores or natriuretic peptides, or to consider specific echocardiographic markers to identify HFpEF [30]. Therefore, despite well‐defined diagnostic criteria, there was a substantial risk of HFpEF underdiagnosis, especially for non HF‐specialists [30]. In line with those results, our study suggests that a systematic use of the H_2_FPEF score could be valuable to improve early detection of patients at highest cardiovascular risk, and timely access to optimal HFpEF management, including specific pharmacologic treatment, non‐pharmacological approaches with exercise prescription and weight management, as well as management of associated comorbid conditions like hypertension, diabetes, AF and chronic kidney disease [5, 12, 31].

### 4.1 Strengths and Limitations

Strengths of this study include its focus on a real‐world geriatric rehabilitation population, for which data are scarce in the HFpEF literature, as well as the systematic comprehensive echocardiographic evaluation combined with simultaneous geriatric assessment. Several limitations need to be mentioned. First, it was a single‐center study in a university clinic, which may limit the generalizability of our findings. Second, only 30% of the eligible patients eventually participated to the study. The most common cause of exclusion was refusal or inability to consent, suggesting an inclusion bias toward patients with a lower grade of frailty. However, since most patients were excluded due to an absence of consent, it was not possible to compare the baseline characteristics of included vs excluded patients to confirm this hypothesis. Third, while the H_2_FPEF score was interpreted according to the published criteria, no invasive hemodynamic confirmation of HFpEF diagnosis was available; likewise, natriuretic peptides were not systematically performed. Fourth, outcome comparisons were performed according to three groups of H2FPEF score severity only; a comparison based on matched groups of patients could have provided more accurate outcome estimation, but the limited size of our cohort did not allow to perform such adjustment. Fifth, geriatric assessment was only based on few standard scores. While MMSE is an important screening tool for cognitive dysfunction, it is insufficient for a comprehensive evaluation of mental status. Furthermore, other metrics of frailty, including gait speed or grip strength, were not systematically performed and could not be included in our analysis. Sixth, although the cause of rehospitalization could be obtained from hospital files, the cause of death could not be ascertained for a majority of very old patients, especially if death occurred outside the hospital. For this reason, it was not possible to segregate the mortality outcome as cardiovascular versus non‐cardiovascular in our cohort. Seventh, some patients underwent rehabilitation following an episode of acute HF decompensation, which may have transiently altered echocardiographic indices of diastolic dysfunction and potentially influenced H_2_FPEF score classification and interpretation in these cases. However, Hullin et al. demonstrated E/e’ remained stable following decongestion in acute HFpEF patients (no significant change between admission and discharge TTE despite 1.8 kg weight loss) [32], supporting the validity of diastolic function assessment in our geriatric cohort. Finally, although follow‐up data were obtained for most patients, some outcomes relied partly on proxy reports, potentially introducing an information bias.

## 5 Conclusion

High‐risk H_2_FPEF score is present in one fourth and a high CCI in 42% of older patients admitted for rehabilitation after acute hospitalization. While both high‐risk H_2_FPEF score and high CCI were independently associated with an adverse cardiovascular outcome at 1‐year follow‐up, the association of both scores in the high‐risk range identified patients with 4‐to‐5‐fold higher risk of mortality or CR at 1 year. A systematic implementation of the H_2_FPEF score in this multimorbid population has the potential to not only to detect patients with a high likelihood of HFpEF diagnosis but also to improve the prognosis with a timely introduction of pharmacologic and non‐pharmacologic management of HFpEF and associated comorbid conditions.

## Author Contributions

**Nicolas Dayer:** conceptualization, data curation, analysis, investigation, methodology, redaction, edition, visualization. **Denise Auberson:** conceptualization, data curation, analysis, investigation, methodology, redaction, edition. **Henri Lu:** conceptualization, data curation, analysis, investigation, methodology, redaction, edition. **Elsa Costa:** data curation, investigation. **Hélène Krief:** statistical analysis, investigation. **Sylvain Nguyen:** conceptualization, methodology, edition. **Kristof Major:** conceptualization, data curation, analysis, investigation, methodology, edition. **Wanda Bosshard:** conceptualization, methodology, edition. **Roger Hullin:** conceptualization, analysis, investigation, methodology, redaction, edition. **Pierre Monney:** conceptualization, data curation, analysis, supervision, investigation, methodology, project administration, redaction, edition, visualization.

## Ethics Statement

This study was approved by the Ethics Committee of the Canton of Vaud, Switzerland (CER‐VD 2021‐01693).

## Consent

Written informed consent was obtained from all included participants.

## Conflicts of Interest

The authors declare no conflicts of interest.

## Supporting information


Supporting File

## Data Availability

The data that support the findings of this study are available from the corresponding author upon request, subject to applicable ethical and privacy restrictions.
