# Supplementary material for: H2FPEF Score and Charlson Comorbidity Index as Independent Predictors of Cardiovascular Outcomes in Elderly Patients Undergoing Geriatric Rehabilitation
Source: Clin Cardiol. 2026 Oct 16;49(10):e70496. doi: 10.1002/clc.70496 (PMC13674404; doi:10.1002/clc.70496)
Supplement: Supplementary file 1 [file CLC-49-e70496-s001.docx]

**Supplementary material S1. H2FPEF score[1]**

| **Clinical or echocardiographic criterion** | **Points assigned** |
| --- | --- |
| BMI ≥ 30 kg/m² (obesity) | 2 |
| Treatment with ≥2 antihypertensives | 1 |
| Age > 60 years | 1 |
| Atrial fibrillation | 3 |
| E/e′ ratio > 9 (echocardiographic) | 1 |
| Pulmonary artery systolic pressure > 35 mmHg (echocardiographic) | 1 |

**Total score: 0–9 points**

- Score ≤ 2: low probability of HFpEF
- Score 3–5: intermediate probability of HFpEF
- Score ≥ 6: high probability of HFpEF (>90%)

**Supplementary material S2. Description of Functional Geriatric Assessment Tools**

**Basic Activities of Daily Living (BADL):[2]** The BADL scale assesses fundamental self-care tasks essential for daily functioning, including bathing, dressing, toileting, transferring, continence, and feeding. It is widely used in geriatric medicine to evaluate physical dependency and identify the need for assistance in core activities. The Katz Index for BADL typically ranges from 0 to 6 points, with higher scores indicating greater independence.

**Instrumental Activities of Daily Living (IADL):[3]** The IADL scale evaluates more complex activities required for independent living, such as managing finances, preparing meals, medication management, shopping, housekeeping, and using a telephone. It complements BADL by providing early detection of functional decline that affects autonomy. The Lawton IADL score ranges from 0 to 8 points, where higher scores indicate greater independence.

**Functional Independence Measure (FIM):[4]** The FIM is a comprehensive tool assessing physical, psychological, and social function via 18 items divided into motor and cognitive domains, including mobility, self-care, communication, and social cognition. It quantifies the level of dependence and is commonly used in rehabilitation settings. The total FIM score ranges from 18 (complete dependence) to 126 (full independence).

**Mini-Mental State Examination (MMSE):[5,6]** The MMSE is a standardized cognitive screening instrument that assesses orientation, attention, memory, language, and visuospatial skills. Scores range from 0 to 30, with higher scores reflecting better cognitive function. It is widely validated for detecting cognitive impairment and dementia. Scores ≤24 suggest cognitive impairment though cutoffs may vary slightly depending on education level and population.

**Tinetti Performance Oriented Mobility Assessment:[7]** The Tinetti score evaluates balance and gait abilities to assess fall risk in older adults. It includes assessments of static balance (e.g., sitting, standing, turning) and dynamic gait components. The combined score ranges from 0 to 28 points, where higher scores indicate better mobility and lower fall risk.

**References**

[1]	Reddy YNV, Carter RE, Obokata M, et al. A Simple, Evidence-Based Approach to Help Guide Diagnosis of Heart Failure With Preserved Ejection Fraction. Circulation 2018;138:861–70. https://doi.org/10.1161/CIRCULATIONAHA.118.034646.

[2]	Katz S. Studies of Illness in the Aged: The Index of ADL: A Standardized Measure of Biological and Psychosocial Function. JAMA 1963;185:914. https://doi.org/10.1001/jama.1963.03060120024016.

[3]	Lawton MP, Brody EM. Assessment of Older People: Self-Maintaining and Instrumental Activities of Daily Living. The Gerontologist 1969;9:179–86. https://doi.org/10.1093/geront/9.3_Part_1.179.

[4]	Keith RA, Granger CV, Hamilton BB, et al. The functional independence measure: a new tool for rehabilitation. Adv Clin Rehabil 1987;1:6–18.

[5]	Tombaugh TN, McIntyre NJ. The Mini‐Mental State Examination: A Comprehensive Review. J Am Geriatr Soc 1992;40:922–35. https://doi.org/10.1111/j.1532-5415.1992.tb01992.x.

[6]	Folstein MF, Folstein SE, McHugh PR. “Mini-mental state.” J Psychiatr Res 1975;12:189–98. https://doi.org/10.1016/0022-3956(75)90026-6.

[7]	Tinetti ME. Performance‐Oriented Assessment of Mobility Problems in Elderly Patients. J Am Geriatr Soc 1986;34:119–26. https://doi.org/10.1111/j.1532-5415.1986.tb05480.x.
